# Cancer Vaccines: Adjuvant Potency, Importance of Age, Lifestyle, and Treatments

**DOI:** 10.3389/fimmu.2020.615240

**Published:** 2021-02-17

**Authors:** Stefania Cuzzubbo, Sara Mangsbo, Divya Nagarajan, Kinana Habra, Alan Graham Pockley, Stephanie E. B. McArdle

**Affiliations:** ^1^ Université de Paris, PARCC, INSERM U970, 75015, Paris, France; ^2^ Laboratoire de Recherches Biochirurgicales (Fondation Carpentier), Assistance Publique-Hôpitaux de Paris (AP-HP), Hôpital Européen Georges Pompidou, Paris, France; ^3^ Ultimovacs AB, Uppsala, Sweden; ^4^ Department of Pharmaceutical Biosciences, Science for Life Laboratory, Uppsala University, Uppsala, Sweden; ^5^ Department of Immunology, Genetics and Clinical pathology Rudbeck laboratories, Uppsala University, Uppsala, Sweden; ^6^ The School of Science and Technology, Nottingham Trent University, Nottingham, United Kingdom; ^7^ The John van Geest Cancer Research Centre, School of Science and Technology, Nottingham Trent University, Nottingham, United Kingdom; ^8^ Centre for Health, Ageing and Understanding Disease (CHAUD), School of Science and Technology, Nottingham Trent University, Nottingham, United Kingdom

**Keywords:** cancer vaccine, adjuvant, immunotherapy, inflamm-aging, microbiota, immunosenescence

## Abstract

Although the discovery and characterization of multiple tumor antigens have sparked the development of many antigen/derived cancer vaccines, many are poorly immunogenic and thus, lack clinical efficacy. Adjuvants are therefore incorporated into vaccine formulations to trigger strong and long-lasting immune responses. Adjuvants have generally been classified into two categories: those that ‘depot’ antigens (e.g. mineral salts such as aluminum hydroxide, emulsions, liposomes) and those that act as immunostimulants (Toll Like Receptor agonists, saponins, cytokines). In addition, several novel technologies using vector-based delivery of antigens have been used. Unfortunately, the immune system declines with age, a phenomenon known as immunosenescence, and this is characterized by functional changes in both innate and adaptive cellular immunity systems as well as in lymph node architecture. While many of the immune functions decline over time, others paradoxically increase. Indeed, aging is known to be associated with a low level of chronic inflammation—inflamm-aging. Given that the median age of cancer diagnosis is 66 years and that immunotherapeutic interventions such as cancer vaccines are currently given in combination with or after other forms of treatments which themselves have immune-modulating potential such as surgery, chemotherapy and radiotherapy, the choice of adjuvants requires careful consideration in order to achieve the maximum immune response in a compromised environment. In addition, more clinical trials need to be performed to carefully assess how less conventional form of immune adjuvants, such as exercise, diet and psychological care which have all be shown to influence immune responses can be incorporated to improve the efficacy of cancer vaccines. In this review, adjuvants will be discussed with respect to the above-mentioned important elements.

## Introduction

**Figure 1:**
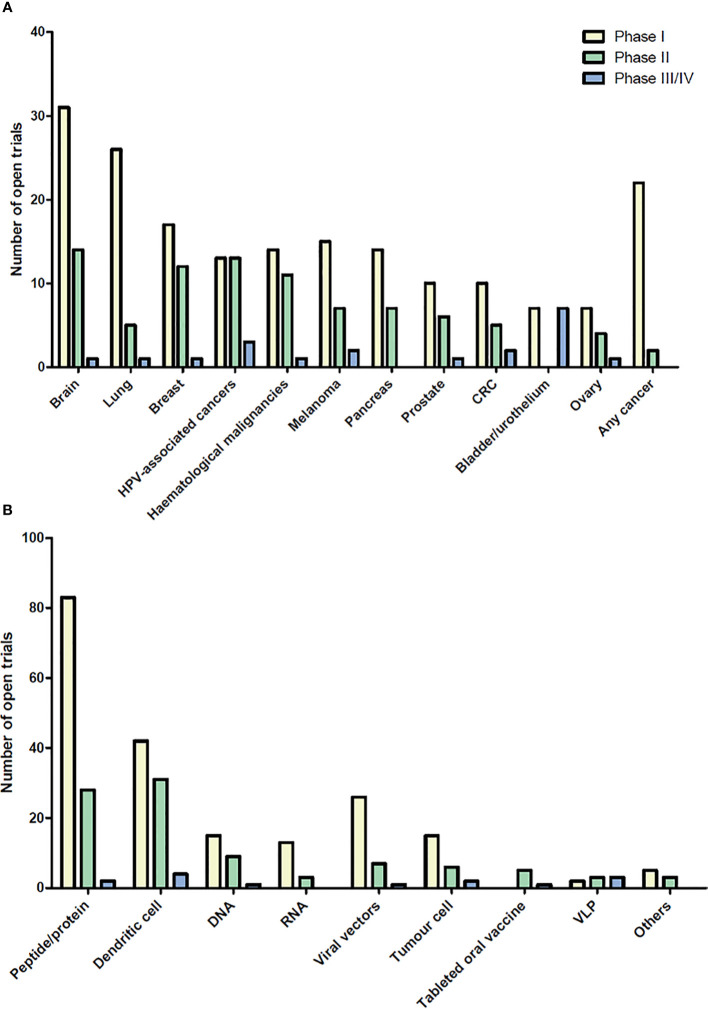
Open cancer vaccine trials. Cancer vaccine trials listed as open at ClinicalTrials.gov on August 2020. The number of trials for each cancer type **(A)** and for each vaccine type **(B)** are shown in the bar graph subdivided into phase I, II, and III/IV. Viral vector vaccines include adenovirus and poxvirus, but also trials using yeast-loaded antigens and one using Salmonella-loaded antigens. Cancers with less than 5 open clinical trials are not shown. “*In situ* vaccinations” (intralesional injection of immune- modulatory molecules) are not included in these graphs. HPV, Human Papilloma Virus; CRC, colorectal cancer; VLP, virus like particle.

**Figure 2:**
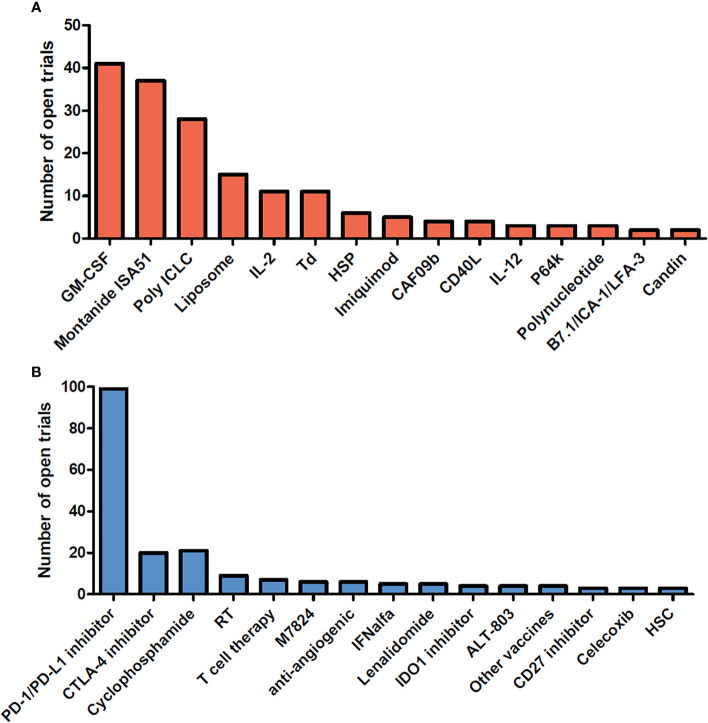
Adjuvants and combinatorial immunomodulatory therapies being used in cancer vaccine trials. Cancer vaccine trials listed as open at ClinicalTrials.gov on August 2020. The number of trials using each adjuvant **(A)** and associating each immunomodulatory therapy with the cancer vaccine **(B)** are shown in the bar graph. Adjuvants and combinatorial therapies used in less than 2 clinical trials are not shown. GM-CSF, Granulocyte-macrophage colony-stimulating factor; IL-2, interleukin-2; Td, Tetanus/diphtheria toxoid; HSP, heat shock protein; CAF09b, cationic liposomes (DDA-MMG1) with complex bound synthetic double-stranded RNA (Poly(I:C)2); IL-12, Interleukin- 12; P64k, Neisseria meningitides protein; PD-1, Programmed cell death 1; PD-L1, Programmed cell death ligand 1; CTLA-4, cytotoxic T-lymphocyte-associated protein 4; RT, radiotherapy; M7824, fusion protein composed of a human IgG1 monoclonal antibody against PD-L1 fused with 2 extracellular domains of TGF-βRII; IFNalfa, Interferon alfa; IDO1, indoleamine 2,3-dioxygenase 1; ALT-803, IL-15 superagonist; Other vaccines, Salmonella, pneumococcal vaccines; HSC, hematopoietic stem cells.

Therapeutic cancer vaccines represent an attractive strategy to stimulate protective anti-tumor immunity in combination with standard therapies. Cumulative data have confirmed the efficacy of cancer vaccines in many murine tumor models, as well as in phase I and II clinical trials. In view of these promising results, numerous clinical trials are ongoing. **Figures 1** and **2** summarize open cancer vaccine trials, distinguished by trial phase, cancer type and vaccine type (**Figure 1**) and by adjuvant and combinatorial treatments used (**Figure 2**). However, cancer vaccines have not yet achieved significant clinical efficacy in phase III trials (**Table 1**). Indeed, clinical responses have been rather anecdotal (48, 49). The reasons for those failed trials are not fully understood but are most likely related to the stage of the disease treated, an inherent difficulty to mount a strong cellular immune response to non-live vaccine entities when older and the choice of antigens, adjuvant and the suppressive nature of the tumor microenvironment. Among these reasons, the difficulty of achieving strong cellular immune responses is likely a major factor to consider. In contrast to prophylactic vaccines against infectious agents that usually trigger humoral responses, therapeutic cancer vaccines aim to promote T cell immune responses for effectiveness. Moreover, very limited considerations have been given to the pharmacokinetic profile of the antigen/adjuvant administration strategy and, consequently, the required durable and effective long-term CD4^+^ and CD8^+^ T cell responses are not achieved. As mentioned, the developed tumor microenvironment is typically immunosuppressive and is characterized by the presence of exhausted T and NK cells and the accumulation of several suppressive immune cells, such as T regulatory cells, T helper type-2 (Th2) CD4^+^ T cells, tumor-associated macrophages (TAMs), and myeloid-derived suppressor cells (MDSCs) (50–53), in addition to which the activation state of T cells will be regulated by co-inhibitory pathways. However, the approval of the first cancer vaccine (Provenge^®^) in 2010 spurred hope (54), as did the reported clinical effects and efficacy of checkpoint inhibitors in some advanced cancer patients. However, global availability of the former is limited as the EMA approval was withdrawn in 2015 (55) and the clinical efficacy of the latter is restricted to a few cancers. Nonetheless, many combination strategies involving immune-based therapies and checkpoint inhibition approaches are currently being tested in phase III trials (**Table 1**, **Figure 2B**). In relation to the suppressive environment, a key factor is to initiate vaccination in tumor indications early on in the disease, non-metastatic, as the lesion size may impact the effectiveness of the treatment (56). This strategy is however hampered by lack of end-points that facilitates studies that fall within a time-frame that can be viable for the industry sponsored clinical trials.

**Table 1:** Completed phase 3 cancer vaccine trials.

Vaccine	Clinical trial (NCT number)	Cancer	Vaccine type	Antigen target	Adjuvant	Associated treatment	Control arm	Results: immunological response(from phase II and III)	Results: clinical efficacy? (from phase III)	References
Peptide vaccine	01989572	Melanoma	Peptide	tyrosinase, gp100, MART-1	Montanide ISA-51 GM-CSF	/	GM-CSF or Placebo	Cell-mediated (CD8)	No (PFS and OS)	(1)
MDX-1379	00094653	Melanoma	Peptide	gp100	Montanide ISA-51	+/- Ipilimumab	Placebo + ipilimumab	NA	No (OS) longer survival with Ipilimumab	(2)
gp100:209-217(210M) peptide vaccine	00019682	Melanoma	Peptide	gp100	IL-2	/	IL-2	Cell-mediated (no correlation with OS)	Yes (PFS and ORR)	(3)
GSK1572932A	00796445	Melanoma	Peptide	MAGE-A3	AS15	/	Placebo	Antibody-mediated	No (PFS)	(4)
GM2-KLH	00005052	Melanoma	Ganglioside	Ganglioside GM2	QS-21 KLH	/	Observation	NA	No (PFS and OS) Detrimental outcome	(5)
CancerVax (CANVAXIN)	00052156 00052130	Melanoma	Allogeneic tumor cell	/	BCG	/	Placebo + BCG	NA	No (PFS and OS) Detrimental outcome	(6, 7)
Melanoma vaccine	01861938	Melanoma	Allogeneic tumor cell (expressing HLA A2/4-1BB ligand)	/	BCG	Cyclophosphamide	/	Cell-mediated (CD8)	Pending	(8)
Melacine	–	Melanoma	Allogeneic tumor cell lysate	/	Detox	/	Observation	Cell-mediated (CD8)	No (PFS and OS) Survival benefit in HLA-A2 and –Cw3 population	(9)
GSK1572932A	00480025	NSCL	Peptide	MAGE-A3	AS15	/	Placebo	Antibody-mediated (100%)	No (PFS)	(10)
Tecemotide (L-BLP25)	00409188	NSCL	Peptide	MUC1	Liposome	cyclophosphamide	Placebo and cyclophosphamide	Cell-mediated (no correlation with OS)	No (OS) Survival benefit in patients who received chemoRT and tecemotide	(11, 12)
rEGF-P64K/Montanide ISA-51	00516685 01444118	NSCL	Protein	EGF	Montanide ISA-51 P64k	cyclophosphamide	Best supportive care	Antibody-mediated	Yes (OS)	(13)
Lucanix TM	00676507	NSCL	Allogenic tumor cells (transfected with TGF-β2)	/	/	/	Placebo	Cell- and antibody- mediated	No (PFS and OS); Positive trend in previously irradiated and early treated patients	(14, 15)
TG4010	00415818	NSCL	MVA (encoding MUC1 and IL-2)	MUC1	IL-2 (encoded by MVA)	Cisplatin and gemcitabine	Cisplatin and gemcitabine	Cell-mediated (CD8)	Pending From phase IIb study part: positive trend (PFS)	(16)
TG4010	01383148	NSCL	MVA (encoding MUC1 and IL-2)	MUC1	IL-2 (encoded by MVA)	Standard chemotherapy and bevacizumab if prescribed	Placebo + standard chemotherapy and bevacizumab if prescribed	Cell-mediated (CD8) (correlation with OS)	Pending From phase IIb study part: longer PFS and OS in patients with low peripheral baseline of activated lymphocytes (CD16+ CD56+ CD69+)	(17, 18)
Racotumomab	01460472	NSCL	Anti-idiotypic antibody	NeuGcGM3	Alum	/	Best supportive care	Antibody-mediated	Pending From phase IIb study part: Yes (PFS and OS)	(19)
BEC2	00003279 00006352 00037713	SCLC	Anti-idiotypic antibody	GD-3	BCG	/	Best supportive care	Antibody-mediated (no correlation with OS)	No (PFS and OS)	(20, 21)
GVAX^®^	00089856	Prostate	Allogenic tumor cells (secreting GM-CSF)	/	GM-CSF	/	Docetaxel and prednisone	NA	No (survival)	ClinicalTrials.gov
Sipuleucel-T/Provenge	00005947	Prostate	DC	PAP	GM-CSF	/	Placebo	Cell- and antibody- mediated	Yes (OS) No significant difference in PFS	(22)
Sipuleucel-T/Provenge	00065442	Prostate	DC	PAP	GM-CSF	/	Placebo	Cell- and antibody- mediated	Yes (OS) No significant difference in PFS	(23)
Sipuleucel-T/Provenge	00779402	Prostate	DC	PAP	GM-CSF	/	Placebo	Pending	Not significant difference in time to biochemical failure Pending for OS results	(24)
DCVAC	02111577	Prostate	DC	/	/	Docetaxel and prednisone	Placebo + docetaxel and prednisone	Pending	Pending	ClinicalTrials.gov
PROSTVAC	01322490	Prostate	Poxviral vector (transfected with PSA and TRICOM)	PSA	TRICOM +/- GM-CSF	/	Placebo +/- GM- CSF	No (no detectable antibody responses to PSA)	No (PFS and OS)	(25, 26)
Intravesical BCG	00002990	Bladder	*in situ* vaccination	/	BCG	/	Different doses of BCG	/	No (OS)	(27)
intravesical BCG	00002490	Bladder	*in situ* vaccination	/	BCG	/	RT or mitomycin C	NA	No survival benefit with RT (versus BCG or chemotherapy)	(28)
Intravesical BCG	01442519	Bladder	*in situ* vaccination	/	BCG	Electromotive mitomycin	BCG alone	NA	Yes (PFS, OS) in BCG+ mitomycin group	(29)
Intravesical BCG	00330707	Bladder	*in situ* vaccination	/	BCG +/- IFN α	/	/	NA	Higher recurrence in patients with CIS, NRAMP1 D543N G:G, and the (GT)n allele 3 genotypes	(30)
GV1001 (TELOVAC)	00425360	Pancreas	Peptide	Telomerase	GM-CSF	Gemcitabine and capecitabine	Gemcitabine and capecitabine	Cell-mediated (CD4)	No (OS) on overall population, approved indication in South Korea using eotaxin levels as biomarker for patient stratification	(31)
HyperAcute-pancreatic (Algenpantucel-L)	01072981	Pancreas	Allogenic tumor cells (expressing murine α -gal epitopes)	/	/	Gemcitabine +/- 5FU chemoradiation	Gemcitabine +/- 5FU chemoradiation	Pending	Pending	(32)
PANVAC-VF	00088660	Pancreas	poxvirus vector (expressing CEA, MUC1 and TRICOM)	CEA, MUC1	GM-CSF	/	Best supportive care or palliative chemotherapy	Cell-mediated	Pending	(33)
FNHLId1	00091676	NHL	Protein	Ig idiotype	KLH GM-CSF	/	KLH + GM-CSF	Cell-mediated (CD4 and CD8)	Yes (PFS)	(34, 35)
FavId (Mitumprotimut- T)	00089115	NHL	Protein	Ig idiotype	KLH GM-CSF	Previous rituximab	Placebo + GM-CSF	Cell- (72%) and antibody- (20%) mediated	No (PFS and ORR)	(36, 37)
MyVax	00017290	NHL	Protein	Ig idiotype	KHL GM-CSF	Previous chemotherapy	KLH + GM-CSF	Antibody-mediated (41%)	No (PFS)	(38)
DCVax-L	00045968	GBM	DC	/	/	/	Placebo	Pending	Pending	(39)
Rindopepimut	01480479	GBM	Peptide	EGFRvIII	KLH GM-CSF	Temozolomide	KLH + temozolomide	Antibody-mediated	No (OS)	(40)
NeuVax	01479244	Breast	Peptide	HER2	GM-CSF	/	Placebo and GM- CSF	NA	No (PFS)	(41)
THERATOPE	00003638	Breast	Peptide	Sialyl-Tn	KLH Detox-B	cyclophosphamide	KLH-vaccine and cyclophosphamide	Antibody-mediated (correlation with OS)	No (PFS and OS)	(42, 43)
HSPPC-96/vitespen	00033904	RCC	Autologous tumor-derived HSPPC-96	/	/	/	Observation	NA	No (PFS)	(44)
IMA901	01265901	RCC	Peptide	TUMAPs	GM-CSF	Cyclophosphamide and Sunitinib	Sunitinib	Cell-mediated (CD8) (no correlation with OS)	No (OS)	(45)
BCG	00427570	CRC	*in situ* vaccination	/	BCG	/	Observation or chemotherapy	NA	Yes (OS) compared to observation	(46)
Gardasil	02087384	Anus	VLP	HPV-6, 11, 16, 18	Alum	/	Placebo	Pending	Pending	ClinicalTrials.gov
Abagovomab	00418574	Ovary	anti-idiotypic antibody	CA-125	/	/	Placebo	Antibody-mediated	No (PFS and OS)	(47)

Phase 3 cancer vaccine trials listed as completed at ClinicalTrials.gov on August 2020. Immune responses results are reported as published in phase III data when available or in phase II respective data of the same vaccine and same authors group.

5FU, 5-fluoruracil; BCG, Bacillus Calmette–Guérin; CA-125, carcinoma antigen 125; CEA, Carcinoembryonic antigen; CRC, colorectal carcinoma; Detox, detoxified Freund’s adjuvant; DC, dendritic cell; EGF, epidermal growth factor; GBM, glioblastoma; GM-CSF, Granulocyte-macrophage colony-stimulating factor; HER2, human epidermal growth factor receptor 2; HSPPC-96, Heat Shock Protein Peptide Complex-96; HPV, human papillomavirus; IL-2, Interleukin-2; Ig, immunoglobulin; KLH, keyhole limpet hemocyanin; MUC1, Mucin 1; MVA, modified vaccinia virus Ankara; NSCLC, non-small cell lung cancer; ORR, objective response rate; OS, overall survival; PAP, Prostatic acid phosphatase; PFS, progression free survival; PSA, Prostate-specific antigen; SCLC, small cell lung cancer; RCC, renal cell carcinoma; RT, radiotherapy; TGF-β2, Transforming growth factor-beta 2; TUMAP, PLIN2, APOL1, CCND1, GUCY1A3, PRUNE2, MET, MUC1, RGS5, MMP7, HBcAg; TRICOM, B7.1 + ICAM-1, InterCellularAdhesion Molecule-1 + LFA-3, Leukocyte function-associated antigen-3; VLP, virus like particle.

Another potentially confounding issue with regards to the efficacy of cancer vaccines is age, given that the median age of cancer diagnosis is 66 years, and the immune system is known to decline with age. This phenomenon, known as immunosenescence, is characterized by functional changes in both innate and adaptive cellular immunity as well as in lymph node architecture. While many of the immune functions decline over time, others paradoxically increase. Indeed, aging is known to be associated with a low, but persistent level inflammation. “Inflamm-aging” also leads to dysregulation of innate and adaptive immune cells (57–59).

It is therefore essential that the choice of adjuvants is carefully optimized for each vaccine formulation, as well as for each patient, in order to break immune tolerance and achieve maximum immune responses and clinical efficacy, even in such a compromised environment. Most cancer antigens are poorly immunogenic and adjuvants are required to (i) prolong the antigen availability at the injection site (“depot” effect); (ii) activate the innate immunity; (iii) direct the immune response toward T helper type-1 (Th1) responses; and (iv) to mitigate the tumor/associated immune suppression (60, 61). Based on function, classical adjuvants have generally been divided into two categories: the immunostimulatory adjuvants (cytokines, Toll-Like receptor agonists, saponins …) and “depot” adjuvants (e.g. mineral salts such as aluminum hydroxide, emulsions, liposomes). Although practical, this classification is today rather simplistic since some delivery systems can also activate innate immunity by creating local proinflammatory reactions (62). Novel RNA-based vaccines have an inherited adjuvant capacity that has also been associated with problematic toxicity, handled by elegant design and formulation (63). As such RNA-based vectors, which have had so far been developed for cancer treatment, are now in a record development program reaching the society in response to the COVID-19 pandemic. This also sets the scene for many novel indications ahead (64, 65).

In this review, adjuvants approved for human use will be discussed with respect to the above-mentioned elements. Importantly, new forms of adjuvants including exercise, microbiota and the psychological status of the patient prior to immunization will also be discussed.

## Immunostimulatory Adjuvants

Immunostimulant adjuvants likely constitute the most promising strategy to potentiate immune responsiveness in elderly cancer patients. Numerous defects in the innate and adaptive immune system have been indeed described in elderly individuals. Age-related reductions in levels of major histocompatibility (MHC) class II expression as well as dysregulation of cellular signaling in human and murine monocytes compromise the efficiency of antigen presentation to T cells (66–68). Studies on peripheral blood mononuclear cells (PBMCs) from elderly donors have also revealed that aged dendritic cells (DCs) have a reduced capacity for producing inflammatory cytokines in response to inflammatory stimuli, and particularly to several Toll-like receptor (TLR) ligands, as well as an impaired ability to present antigens to T cells (59, 69–71). Alongside defects in innate immune potential, numerous reports have described the phenomenon of T cell immunosenescence, an event which primarily results from thymic involution which leads to a contraction of the naïve T cell compartment and a predominance of terminally differentiated memory T cells in the periphery (72, 73). Other studies suggest that chronic latent infections, such as cytomegalovirus (CMV), could also play a crucial role in T cell immunosenescence in the CD4^+^ T cell compartment, as well as in the naïve and memory CD8^+^ T cell compartments (74, 75). Permanent CMV infection stimulates the expansion of CMV-specific memory CD8^+^ T cells and could thus impact on the ability of an individual’s T cells to elicit a response against new antigens (76). Additionally, the chronic inflamm-aging status observed with age has been associated with diminished expression of the costimulatory receptor CD28 on T cells because of persistently increased levels of TNF-α (77–79). CD28 is vital for efficient T cell activation, reduced levels of which have been correlated with poor immune responses after vaccination among older people (80, 81).

### Cytokines

The use of cytokines in cancer immunotherapy and specifically in cancer vaccine formulations is becoming more prevalent as they can elicit both cellular and humoral immune responses. IFN-α, IFN-γ, IL-2, IL-12, IL-15, IL-18, IL-21 have especially demonstrated immunological efficacy when used as part of a vaccine adjuvant strategy (50). However, to date, GM-CSF (granulocyte macrophage colony-stimulating factor) is the immunostimulatory cytokine which has been most widely used in clinical vaccine trials (**Figure 2A**). GM-CSF has been reported to induce strong T cell responses as well as to inhibit tumor growth in both whole tumor cell and peptide vaccines in preclinical studies (82) by recruiting and activating antigen-presenting cells (APCs) at the injection site. However, GM-CSF as vaccine adjuvant has delivered conflicting results in clinical trials. In some trials, GM-CSF has shown only weak effects in potentiating immune response of cancer vaccine (83, 84) and in others no additional positive effect was reported when associated with Montanide (85, 86). However, the only FDA approved cancer vaccine, Provenge^®^, bases the adjuvant effect on a fusion protein that contains GM-CSF and reported an OS benefit in patients with metastatic castrate-resistant prostate cancer. However, in phase II and III trials testing Provenge^®^, the exact role/influence of GM-CSF over clinical efficacy was not thoroughly investigated. In addition, two trials containing GM-CSF in the vaccine formulation resulted in decreased cell-mediated immune responses and shorter survival of patients with melanoma (87, 88), however it is also possible that in this instance the choice of antigen as well as formulation have had an impact on the results. Indeed, lower doses of GM-CSF in water formulations could shape the lymph node differently compared to a montanide based formulation. Interestingly, daily doses of GM-CSF over 100 µg/day given repeatedly have been reported to promote the expansion of myeloid-derived suppressor cells (MDSCs) and inhibit T-cell function (89). While GM-CSF as an adjuvant in prime/boost administration given at lower doses has shown good adjuvant capabilities and is an adjuvant commonly used in many vaccine formulations due to the expanded knowledge of the adjuvant (**Table 1**, **Figure 2A**).

Systemic use of cytokines such as IL-2 or GM-CSF in combination with other immunotherapies have also shown clinical efficacy (3, 90). It is therefore extremely important to optimize the schedule, formulation and the dose of cytokine in order to avoid/limit systemic side-effects.

### Toll-Like Receptor Ligands

The stimulation of professional APCs such as neutrophils, B cells, macrophages and DCs is an efficient approach to boost the efficacy of cancer vaccines, especially in immunocompromised individuals, such as cancer patients and more generally the elderly. However, as indicated above, age-related deficiencies in monocyte and macrophage function mediated by functional dysregulation of cellular signaling, and specifically of Toll-like receptor (TLR) pathway have been described. Activation of APCs relies upon stimulation of pathogen recognition receptors (PRRs) by conserved pathogen-associated molecular patterns (PAMPs) expressed on microbes, or endogenous danger-associated molecular patterns (DAMPs) released by injured cells. TLRs recognizing PAMPs and DAMPs under physiological conditions are expressed either on the cell membrane (TLR1, -2, -4, -5, -6, and -10) or on endosomal membranes within the cell (TLR3, -7, -8, and -9) according to the ligand - membrane TLRs bind lipids and proteins whereas intracellular TLRs bind nucleic acids (91, 92). PRR activation induces the release of chemokines and inflammatory cytokines, the recruitment of innate and adaptive immune cells, and stimulation of the APCs themselves *via* the induction of costimulatory molecule expression, including B7.1 (CD80), B7.2 (CD86) and CD40.

Studies on peripheral blood mononuclear cells (PBMCs) from elderly donors have shown that that aged DCs have a diminished ability in producing cytokines in response to inflammatory stimuli, and particularly to TLR1/2 and TLR7/TLR9 ligands, as well as an impaired capacity for presenting antigens to T cells (69–71). Such deficiencies have been associated with a decreased activity of PI3K that results in aberrant activation of NF-kB and therefore a weak, but chronic inflammatory state characterized by continuous release of IL-6 and TNF-α cytokines (93), the so-called phenomenon of inflamm-aging. The overall result is a compromised ability of DCs to orchestrate an efficient adaptive immune response in elderly individuals (59).

In view of these defects on DCs, TLR ligands which mimic PAMPs represent promising adjuvant candidates for cancer vaccines in elderly individuals. Synthetic TLR3, TLR7 and TLR9 agonists are likely the best candidates, as they mimic viral RNA and DNA PAMPs (94) which generally generate robust cytolytic CD8^+^ T-cell responses (95, 96). Specifically, TLR3 recognizes viral dsRNA and their synthetic analog Poly I:C (97, 98); TLR7 binds viral ssRNA, whereas TLR9 interacts with unmethylated CpG DNA from bacteria and viruses (91, 92). Three TLR ligands are FDA-approved for cancer therapy: Bacillus Calmette-Guérin (BCG), a TLR2/4 ligand, the TLR4 ligand monophosphoryl lipid A (MPLA) and the TLR7 agonist imiquimod. However, many other TLR agonists have proven their efficacy in pre-clinical and clinical studies.

The use of TLR agonists constitutes an efficient way to boost the efficacy and potency of cancer vaccines thanks also to their interaction with other immune and non-immune cells which can express TLRs, including T-cells and cancer cells. Indeed, poly I:C (TLR3 agonist) has been reported to stimulate the proliferation and survival of both CD4 and CD8 T cells in a CF-kB-dependent manner (99, 100). Additionally, in human CD4^+^ Th cells, the stimulation of TLR7/8 and TLR5 by resiquimod and flagellin increases IFN-γ, IL-2, and IL-10 release and enhances proliferation in an APC-independent manner (101). Other studies have shown similar effects of TLR9 stimulation on the survival and proliferation of CD4^+^ and CD8^+^ T-cells. This effect was mediated by NF-kB signaling and was associated with increased expression of the anti-apoptotic protein Bcl-xL (99). Furthermore, TLR9 stimulation of CD4^+^ T-cells can render them resistant to the immunosuppressive effects of regulatory T cells (Tregs) (102, 103).

Beside APCs and T cells, TLRs are also expressed by a multitude of cancer cells. Their direct effect on cancer cells is not completely defined, and probably much less important than their immune effects. The activation of TLR2 and TLR4 in cancer cells has been linked to tumor-promoting effects by promoting vascularization and cell invasion *via* the induction of COX-2, PGE2 and IL-8 (104, 105). Similar to TLR2 and TLR4, TLR7/TLR8 overexpression in lung cancer cells has been associated with pro-tumor effects through the activation of NF-kB and resulting in upregulation of inflammatory cytokines, the anti-apoptotic Bcl-2, the angiogenic VEGFR2 and several chemokine receptors associated with cell migration (106). TLR3 stimulation by Poly I:C or BCG has been implicated in promoting tumor cell death in a multitude of cancers, including breast cancer, colon cancer, bladder cancer, head and neck carcinoma, pharynx carcinoma, hepatocellular carcinoma, lung cancer and melanoma. TLR3 polymorphisms have also been linked to an increased risk of several cancers such as nasopharyngeal carcinoma, breast cancer, cervical cancer, and Hodgkin’s disease (107). However, TLR3 activation has been reported to induce cancer progression as well by the induction of VEGF, MMP9 and uPAR *via* Myc- and MAPK signaling (108). TLR5 signaling on cancer cells has been reported to inhibit tumor growth in various cancers, including breast cancer (109), head and neck cancer (110) and colon cancer (111). On the contrary, TLR5 stimulation in gastric cancer cells, notably by H. pylori, has been reported to increase IL-8 production, tumor cell proliferation as well as TNF-α expression levels that can support the suppressive effects of Treg cells (112, 113). Depending upon tumor cell types, TLR9 activation can stimulate or inhibit tumor cell proliferation (114–118) or induce caspase-dependent apoptosis (119, 120). The TLR9 agonist CpG-ODN has proved to be moderately effective in glioblastoma patients when injected intratumorally (121, 122).

In summary, various TLRs can be expressed on numerous cancer cell types and TLR3 and TLR5 appear to be the most promising adjuvants for combining direct anti-tumor properties with immunostimulant effects on APCs and T cells. However, the final effect of each TLR agonist relies on its immunostimulant properties. Therefore, the choice of the TLR agonist should be primarily driven by its ability to trigger T cell response in humans, which should be defined on a case-by-case basis for a given antigen.

### Saponins

Saponin adjuvants are extracts from the plant *Quillaja saponaria* and possess potent inflammatory properties. QS-21 is the most commonly used adjuvant in vaccine formulations (123) and its immunogenicity has been attributed to the triterpene aldehyde group, which is capable of triggering the ASC/NALP3 inflammasome signaling and thus stimulating the conversion from precursor to activated forms of IL-1 β and IL-18 (124). The adjuvant QS-21 has been reported to elicit robust T-helper 1, CD8^+^ T cell and humoral responses in preclinical studies. Besides such immunogenic properties, QS-21 strongly activates the inflammasome thus causing cell membrane lysis and apoptosis of APCs (124). QS21 has also been tested in clinical trials, mostly as adjuvant of cancer vaccines targeting ganglioside antigens and, despite strong humoral responses, no significant cellular immune responses were observed. Its efficacy in cancer vaccine appears thus limited.

QS-21 has also been used as part of more complex vaccine formulations combining multiple adjuvants, for instance ISCOMATRIX incorporating the saponin adjuvant with antigens in a micellar structure (125), and AS01 and AS15 combining QS-21 with MPL (126).

### Stimulator of Interferon Genes Agonists

Stimulator of Interferon Genes (STINGs) are transmembrane proteins that induce a robust Type I IFNγ response upon activation and are expressed at the highest levels by T cells. STING activation can lead specifically to T cell apoptosis since DCs or macrophages do not exhibit such sensitivity (127). STING agonists are combined with adjuvant systems that specifically target myeloid cells (128) and are capable of reprogramming MDSCs towards a DC-like phenotype expressing IL-2 and co-stimulatory molecules (129). However, differential binding properties of these agonists to human and murine cells poses a challenge for the development of clinical strategies.

Ideally, implementation of STING agonists in cancer vaccines should be combined with potent adjuvant/delivery systems such as liposomes or polymeric nanoparticles or inorganic materials to minimize systemic dissemination that can cause toxic cytokine storm and limited bioavailability.

Currently, ADU-S100 and MK-1454 are being tested along with immune checkpoint inhibitors (ICIs) in early phase clinical trials in patients with advanced/metastatic solid tumors or lymphomas (NCT03172936, NCT03010176). Both require accessible lesions for intratumor injections to avoid systemic toxicity.

## Delivery System as Adjuvants

The classical classification of delivery systems and immunostimulant adjuvants is practical, but not dichotomic since several adjuvants can act as a delivery platform for antigens, while also having some immunomodulating properties. Adjuvants traditionally classified in this category mainly act by improving antigen stability, preventing antigen degradation and finally optimizing its processing and presentation to T cells. The most important delivery system adjuvants and their mechanisms are described below.

### Mineral Salts

Alum is by far the most used adjuvant in approved human vaccines against various infectious organisms (130). Aluminum-based adjuvants are traditionally classified as a delivery system type because their depot effect at the injection site leads to a slow release of the antigens. However, recent reports showed that alum is also capable of stimulating the innate immune response by activating the NLRP3/NALP3 inflammasome complex and triggering the release of uric acid (131, 132).

The adjuvant effect of alum in vaccines against infectious agents essentially results from an induction of a sustained Th2 response, as characterized by antibody production, but generally fails to mount a strong cellular (Th1)-based immune responses that are necessary for robust protective anti-tumor immunity. Hence, the use of aluminum-based adjuvants in cancer vaccines is of limited use (130). However, studies have been able to show that alum can also induce a cytotoxic immune response (133), as well as a clinical efficacy in cancer patients in terms of survival (racotumomab-alum vaccine directed against NeuGcGM3 tumor-associated ganglioside) (19). In addition, recent studies have shown that alum can elicit robust immune responses and have anti-tumor efficacy (in terms of inhibited tumor growth and prolonged survival) when used in nanoscale (134). Nano-aluminum adjuvants can indeed carry more antigens and more efficiently present them to antigen-presenting cells (APCs) in lymph nodes compared to traditional aluminum salt adjuvants which tend, instead, to remain at the injection site because of their positive charge and large particle size (135, 136).

### Emulsions

Emulsions are typically classified as water-in-oil (W/O) or oil-in-water (O/W) formulations and mainly act as delivery system of antigens in the injection site, thereby allowing a slow and prolonged release of the latter. Nevertheless, they also have some immune adjuvant properties by inducing local inflammation and promoting the recruitment of APCs as well as their phagocytic uptake of antigen (137–139).

Complete Freund’s adjuvant (CFA) was the first water-in-oil emulsion to be developed (1930). CFA is a highly potent adjuvant which contains heat-killed mycobacteria but induces a strong local inflammation that often leads to ulceration at the site of injection. Given its adverse effects, CFA is not permitted for use in humans.

Incomplete Freund’s adjuvant (IFA) is also a W/O emulsion, but without mycobacteria. IFA induces more manageable adverse events than CFA and is the “golden standard” of this group of adjuvants for assessing the immunogenicity of antigens in mice. IFA has proven to induce both cellular and humoral immune responses (140–143) and its human equivalent, Montanide ISA-51, has been and continues to be widely used in peptide cancer vaccine formulations in many trials (melanoma, renal carcinoma) (144, 145) (**Figure 2A**). Other studies, however, have shown negative effects of IFA and, more generally, of all W/O emulsions. The slow persistent release of the antigen coupled with the local inflammation induced by the emulsion itself can actually result in the sequestration of primed CD8^+^ T cells at the injection site, when using short peptides, leading to limited T cell homing to the tumor and T cell tolerance (146–149). In addition, W/O emulsions are usually associated with Toll-Like receptor (TLR) agonists and numerous studies have reported a detrimental effect of W/O emulsion on T cell responses triggered by TLR agonists (150).

MF59 is an O/W squalene-based emulsion that is currently licensed for human influenza vaccines (151, 152). As for other adjuvants historically included in this group, MF59 also appears capable of triggering cellular and humoral responses. Indeed, MF59 can promote leukocyte recruitment by inducing macrophages and dendritic cells to secrete several chemokines. MF59 has proven to be effective in elderly subjects in human trials and is currently used in a flu vaccine for individuals > 65 years. The use of MF59 is limited in cancer vaccine strategies because of the primal Th2 response. However, in combination with CpG ODN (cytosine guanine dinucleotide oligodeoxynucleotides, TLR9 agonist), MF59 has proven to induce effective anti-tumor responses in several murine cancer models (94, 153).

### Liposomes

Liposomes are phospholipid vesicles which are used as delivery carriers for antigen or also immunostimulatory adjuvants (154, 155). Allison and Gregoriadis, in 1974–1976 innovated the liposomes and since then all their derivative nanovesicles have become important delivery systems for vaccines. Positively charged liposomes have been reported to trigger more potent immune responses compared to negatively charged liposomes. This efficacy is attributed to both more efficient phagocytosis of positively charged liposomes by APCs (156) and reduced lysosomal degradation of antigens because of a higher pH (157). The key advantages of liposomes are their versatility, plasticity, biocompatibility, and biodegradability. Various choices for the composition and preparation can be achieved from a selection of lipids to target the desired charge, size, distribution, traveling and location of antigens or adjuvants for cancer vaccines (155). However, using liposomes for human applications is restricted due to the lack of stable manufacturing of vaccine-grade liposomes and their high cost (155, 158). To resolve these obstacles in co-formulation, a manufacturable, synergistic anionic liposome platform with TLR4/TLR7 agonists ready for use in clinical trials has been developed (159).

Many animal models using liposomes as delivery agents have shown that liposomal cancer vaccines have superior efficacy over the non-liposomal vaccines (158, 160, 161). In mice challenged with Lewis lung carcinoma cells, liposomal vaccines combining basic fibroblast growth factor and the adjuvant monophosphoryl lipid A (MPLA) induced tumor-specific antibodies and Th1-type immune responses (160). Liposomal delivery of the lipid antigen α-galactosylceramide induced anti-tumor immunity that was protective against lung metastases in 65% of B16 F10-tumor-bearing mice, by activating the NKT cells in the spleen (161). Park et al., developed a peptide-CpG-liposome complex vaccine which was proven to efficiently elicit humoral responses (anti-hTM4SF5 antibodies) and inhibit cancer growth in various murine tumor models (pancreatic cancer, metastatic hepatocellular cancer, colon cancer, lung metastasis model) (162). Liposomal vaccines have also been reported to elicit strong cytotoxic T lymphocyte (CTL) responses against tumor-associated peptides, as in the case of Lip-DOPE-P5-MPL, where the P5 peptide was encapsulated in a complex of 5 lipids (DMPC, DMPG, cholesterol, DOPE and MPLA) conjugated with maleimide-PEG2000-DSPE (163). In a mouse model of neuroblastoma, liposomal delivery of CpG ODNs has been shown to elicit potent anti-tumor effects, whereas the CpG-alone group failed (119). Liposomes were also proven to increase the uptake and stimulation of APCs leading to anti-tumor efficacy when used as delivery system of DNA or RNA complexes in mice (164). Recently, a novel lipopolyplex vector (multi-LP) was proposed for the *in vivo* delivery of mRNA by incorporating the immune adjuvant α-galactosylceramide (α-GalCer) and a multivalent cationic lipid to target the dendritic cells (DCs) without cell-specific functionalization or ligands (165).

In addition to the above, several clinical trials using liposomes as carrier system for vaccine have reported safety, capability of inducing prolonged antigen-specific CD4^+^ and CD8^+^ T cell responses, as well as prolonged survival in various cancers, including non-small-cell lung carcinoma (NSCLC) (166), melanoma (167, 168), follicular lymphoma (169) ovarian (170), breast and prostate cancers (171).

In conclusion, liposomes are versatile delivery systems which can load antigens, proteins, peptides, nucleic acids, and carbohydrates, as well as for the formulation of new types of vaccines targeting the lymphatic system or specific APCs such as macrophages or DCs.

### Virosomes

Virosomes are spheres of natural or synthetic phospholipids (liposomes) incorporated into which are virus envelope phospholipids and viral spike proteins. They were identified in 1975, but the first virosome-based vaccine in humans was Inflexal V for influenza in 2009 (158). Virosome-based vaccines are currently commercialized as preventive vaccines for HPV16 and 18-related cancers (Cervarix™ and Gardasil^®^) (172, 173). Virosomes were widely utilized in cancer vaccines because they are incapable of replicating and therefore are not infectious but retain the ability of the parenting virus while carrying tumor-specific antigens to the APCs to induce immunity (174). Thus, virosomes increase the tumor-specific antibody and T cell responses (175, 176), as has been observed in phase I clinical trial on metastatic breast cancer patients (177, 178). The main advantages of virosomes as efficient prophylactic and therapeutic agents are tissue targeting, immune activation and potentiation. Application of virosomes in cancer vaccine will open a new prospective with multiple safe advantages as a unique delivery system (179–181). Recently, adding magnetic agents to HA-virosomes has been proposed as a ground-breaking innovative platform for treating cerebral tumors by enabling targeting using an external magnetic field from a magnetic helmet (182).

### Nanoparticles

Nanoparticle carriers have the advantage to specifically target the APCs by various formations and strategies. The main types of nanoparticle adjuvants under development include metals, carbon nanotubes and polymers.

*Metallic nanoparticles* have various advantages over polymers and liposomes thanks to their multifunctional properties such as their small particle size, superparamagnetic properties and biocompatibility. Metallic nanoparticles such as γFe2O3, Al2O3, TiO2, ZnO, and SiO2 enhance immune responses mainly by acting as antigen carriers that deliver directly to APCs. Specifically, γFe2O3 with a positive surface charge can be absorbed by proteins with negative charge, promote the immune response and enable labeling and tracking cells at the same time. Enhancing the cross-presentation ability of DCs and T cell activation confers great potential on superparamagnetic iron oxide nanoparticles as adjuvants. However, the mechanisms are still not well defined (183). Gold nanoparticle platforms have been more widely applied in tumor models, challenges with regards to approval from the Food and Drug Administration (FDA) remain a challenge for translating these into the clinical setting (184). Recently, gold nanoparticle surfaces were coated with high cargo density of polyelectrolyte multilayers or peptides to promote the antigen-specific T cell response (184, 185).

*Carbon nanotubes* are extensively used in cancer therapeutics due to their large surface area and good conjugation and encapsulation properties. In the field of cancer vaccines, carbon nanotubes have been especially proven to enhance the embryonic stem cell-based cancer vaccine response in murine colon cancer model (MC38) (186). Despite the encouraging results in pre-clinical studies, the use of carbon nanotubes in humans has been hampered by their potential toxicity. Conflicting data is indeed reported on carbon nanotubes biocompatibility and biodegradability (187).

Although *polymeric particles* have been used in product development for several decades, they have not until relatively recently been considered for vaccine development. However, PLGA (poly lactic-co-glycolic acid) nanoparticles have now been approved for human use by the FDA and the European Medicines Agency (EMA) after being considered as the most nontoxic and slowly degraded vaccine delivery system (188) for target-specific and controlled delivery of drugs, peptides, proteins, antibodies and genes in cancer. Linear polyethyleneimine was recently developed for chemical coupling of protein/peptide ligands to form nano-polyplexes with plasmid DNA or RNA which deliver the nucleic acids into the targeted cells without associated toxicity to healthy cells (189). The delivery of DNA and mRNA using such an approach has a number of advantages, including being safer alternatives to viral vectors, colloidal stability (190, 191) can be exploited using injection-free gene delivery systems (192–194), and the ability to modify with targeting moieties like mannose (195).

In addition to the main nanoparticles above-mentioned, other promising nanoparticles are under development as adjuvants in cancer vaccines. For instance, to enhance the tumor penetration capability, positively charged nanoparticles based on the most abundant polysaccharide in nature (chitosan) have been developed over two decades of research on very complex optimized systems. Also, synthetic melanin nanoparticles have been reported to be an innovative adjuvant for cancer vaccines, in that they efficiently localize to draining lymphoid tissues and exhibit strong immunostimulant properties when loaded with both short and long peptides in mice (196). A melanin-based vaccine in combination with a TLR9 has also proved to be a strong anti-tumor efficacy in cancer murine models and compares favorably with the classical formulation of IFA and TLR9 agonist (197).

Current nanoparticle-based strategies in cancer vaccination and immunotherapy vary. Therapeutic nanomaterials enhance the efficacy of cancer vaccines by increasing the lymphatic delivery of specific antigens or by combining targeting approaches with stimulating materials to synergize and/or modulate immune activation. Primarily, the nanocarriers load the adjuvants by hydrophobic or electrostatic interactions which elevate the immunogenicity of tumor antigens (198). The potential co-delivery of antigens and adjuvants such as TLR ligands to DCs can boost the induction of protective anti-tumor immunity. Thus, the therapeutic cancer vaccine becomes essentially a nano-package of antigen, adjuvant and nano-carriers. For instance, the aliphatic polyesters PLGA and poly-ϵ-caprolactone (PCL) have proven to be efficient vectors for increasing their uptake by DCs due to their critical size, surface charge, surface functionalization and route of administration (199, 200). However, the efficacy of such an approach was proven in minimal residual disease conditions instead of the typical clinical condition of large bulky tumors. The co-delivery of adjuvants with nano-based formulations enhances the cross-presentation and/or skews the immune responses to the desired CD4^+^ T helper phenotypes. Specifically, cancer nano-vaccines co-deliver peptides and TLR9 agonists (201, 202), and gold nanoparticles the anionic TLR3 agonist poly I:C co-delivered with cationic antigen peptides (185). In addition, nanoparticles can support a combinational use of adjuvants to permit exploitation of synergy among certain TLR agonists (159, 203–205). A significant therapeutic example in a late-stage murine melanoma model has been combining the peptide epitope of tyrosine-related protein 2 (Trp2) and CpG-based nano-vaccine with siRNA against TGF-β, which is one of the major cytokines responsible for induction and maintenance of an immunosuppressive tumor microenvironment (206). Effective cross-presentation was promoted using pH-sensitive delivery systems that retain their cargo under the physiological conditions and release antigens in the endosomal microenvironment (~pH 6) (207, 208). Alternatively, an oxidation-sensitive polymersome can respond to the oxidative environment of endosomes to trigger the delivery of antigens and adjuvants in the cytosol (209). Furthermore, modification of liposomes with a cell-penetrating peptide or gold nanoparticles with tumor antigens has also been shown to promote cross-presentation (210, 211).

Although several vaccination strategies have been tested *in vivo*, therapeutic benefits remain mixed and a huge gap between material research, preclinical experimentation and clinical reality remains. Further research into the use of PLGA are warrantied to bridge this gap (199). The delivery of whole-cell cancer vaccines has been accomplished using a PLGA matrix containing tumor lysate as the source of tumor antigens, granulocyte macrophage colony-stimulating factor (GM-CSF) for recruitment of DCs *in situ*, and CpG for DC activation. This PLGA matrix elicited antigen-specific CD8^+^ T cells and increased both prophylactic and therapeutic anti-tumor efficacy (212). Alternatively, plasma membranes of tumor cells have been extracted and coated onto polymeric nanoparticle cores along with the TLR4 agonist MPLA as a tumor cell-mimicking cancer vaccine (198).

Although targeted delivery to DCs and the induction of CD8^+^ T cell responses can be achieved using nano-vaccines consisting of CD40 Ab-modified nanoparticles (213), the efficiencies of DC-targeting and induction of adaptive immune responses require optimization as different DC subsets have characteristic sites of tissue residence, receptor expression profiles and functions (214). Moreover, targeting distinctive tissue sites such as murine lymphoid tissue-resident CD8^+^ DCs and, for human, CD141^+^BDCA-3^+^ DCs and Langerhans cells requires further study for nano-vaccines (215, 216). The efficient draining of nanoparticle carriers to lymphoid tissues has been qualitatively and quantitatively demonstrated using fluorescent or contrast agent-based imaging. For example, a polyester nanoparticle system loaded with ovalbumin (OVA) has been labeled with a near-infrared probe (216), and PLGA nanoparticles have been designed to carry iron oxide particles conjugated with fluorophore-labeled peptide antigen (217). Additionally, synthetic melanin nanoparticles have efficiently localized to the draining lymphoid tissues and have potent immunostimulant properties when loaded with short or long peptides in murine models (196, 197). Delivery systems having different particle sizes composition, morphology, and surface chemistry of particles are promising candidates to be translated into clinics to confirm delivery to the draining lymphatics (218).

### Novel Biomolecule-Based Targeting Strategies

To induce tumor immunity, Fc gamma receptor (FcgR) targeting strategies coupled with antigens have been explored for the purpose of activating both CD8^+^ and CD4^+^ T cells. FcgR cross-linking to improve T cell priming can be achieved *via* the formation of immune complexes *in vivo* (219). For vaccine purposes, the conjugating of a universal tetanus-derived synthetic peptide (minimal tetanus toxin epitope, MTTE) with viral or tumor derived antigens - also in the form of synthetic peptides - can facilitate immune complex formation and FcgR cross-linking which results in DC and T cell activation (220, 221). Alternative strategies to target DCs include fusion strategies based on IgG scaffolds that introduce antigen epitopes in the CDR region and vaccine delivery using a DNA based vector system with the aim to target the high affinity FcgRI (222, 223). The goal and advantage of these technologies is to provide a single drug entity which can harness both the adjuvant and targeting of the antigen to APCs, as well as the potential antigen half-life extension that the methodology provides. It also ensures that HLA/peptide off-rate is not the determining factor for antigen delivery to the lymph node. The challenges presented by these approaches are the species differences in the receptor and immunology biology as well as the costly production of, for example, antibody-based therapies, if used as such.

## Combinatorial Strategies to Improve Cancer Vaccine Efficacy in the Immunosuppressive Context of Cancer Patients

### Combining Different Adjuvants to Induce More Extensive Immune Responses

**Figure 3:**
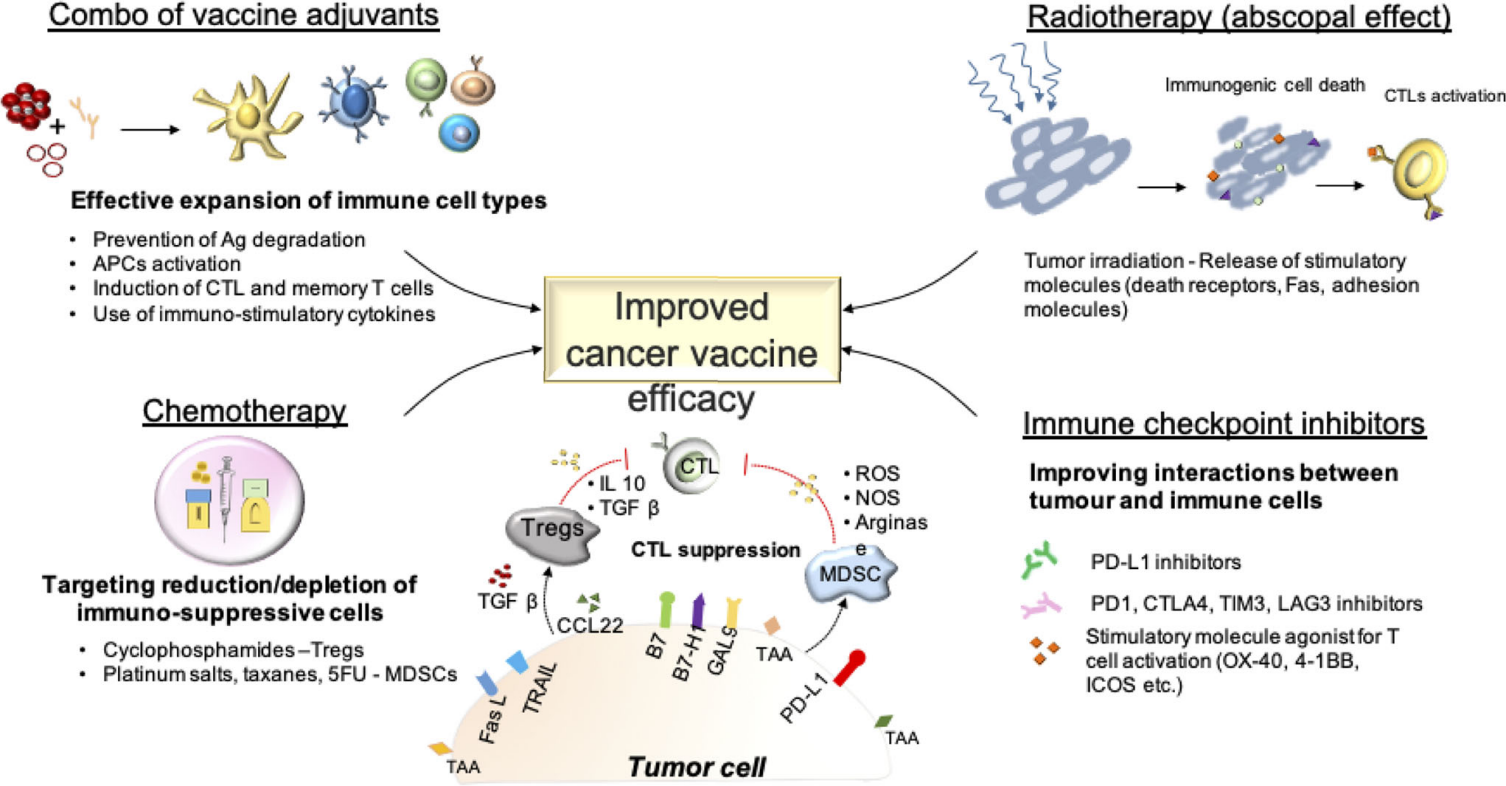
Improving the efficacy of cancer vaccines: Combinational approaches.

The ideal adjuvant for a cancer vaccine formulation should (i) protect antigens from degradation, (ii) stimulate efficient uptake of the antigens by APCs, (iii) activate these APCs to efficiently present the antigen to T cells in order to trigger a strong Th1/CTL response and long-term memory T cells. One single adjuvant may not provide all of these effects at the same time. Thus, a combination of a delivery system adjuvant and an immunostimulant adjuvant is commonly chosen for cancer vaccine formulation (**Figure 3**). For instance, montanide (for depot effect) and a TLR ligand (for APC stimulation) constitutes a common combination of adjuvants for anti-cancer vaccine (224). Based on preclinical studies, combining several TLR agonists or anti-CD40 antibodies with a TLR ligand could potentiate the adjuvant effect by activating different APCs simultaneously and further inducing more extensive CD8^+^ T cell responses (225–227). However, the realization that formulations and the design of the antigen can negatively influence the expansion of a systemic immune response is of importance and should trigger in depth characterization of both the design and physiochemical properties of vaccine components, and the pharmacokinetic profiles and administration dose and schedule, to achieve proper anti-tumor responses.

Another strategy to enhance the efficacy of cancer vaccines could be combining it with systemic immunostimulatory agents such as cytokines, especially IL-2 or GM-CSF. Systemic IL-2 in combination with gp100 peptide-vaccine in patients with melanoma has delivered significant efficacy in terms of objective response and progression-free survival compared to IL-2 monotherapy. However, at such high doses, IL-2 caused numerous toxicities if not formulated correctly (3).

### Blocking VEGF to Restore Tumor Vessels and Promote T Cell Homing to Tumors

Following the induction of peripheral immune response by cancer vaccine, specific anti-cancer T cells need to penetrate the tumor to attack cancer cells. Unfortunately, the tumor vasculature is reported to express reduced levels of leukocyte adhesion molecules, such as intercellular adhesion molecule-1 (ICAM-1) and vascular cell adhesion molecule-1 (VCAM-1), and an aberrant overexpression of immune checkpoints including PD-L1, the death receptors FasL and TRAIL, and IDO, all of which impede the infiltration and function of activated T cells into/in the tumor microenvironment (228).

Anti-angiogenic treatments such as bevacizumab (vascular endothelial growth factor—VEGF—inhibitor) have been reported to restore a normal vasculature within tumors and increase the expression levels of ICAM-1 (229). Additionally, VEGF has also been proven to inhibit T cell and DC activation (230, 231). Therefore, combining an anti-angiogenic treatment such as bevacizumab (VEGF inhibitor) with vaccine seems a valid strategy to enhance the anti-cancer T cells (triggered by the vaccine) homing to tumor. Other molecules such as all-trans retinoic acids, anti-inflammatory triterpenoid, tyrosine kinase inhibitors (sunitinib), IL-12 and anti-IL-6R antibodies, anti-CSF-1R, anti-CCL2 have been reported to reduce tumor infiltration by MDSCs and improve the efficacy of cancer vaccines (232–237). Finally, STING agonists possess the ability to convert MDSCs from a suppressive into a type-1 immune profile (129).

### Depletion of Immunosuppressive Leucocyte Populations by Combining Chemotherapy With Cancer Vaccines

Chemotherapy has long been considered to conflict with immunotherapies due to its leucocyte depleting effect. However, several peripheral and intratumoral leucocyte populations have immunosuppressive properties, thus reducing the efficacy of tumor-reactive cytotoxic T lymphocytes CTLs. A therapeutic strategy could thus rely on combining therapeutic cancer vaccine with leucocyte-depleting chemotherapeutics that target such populations.

Regulatory T (T reg) cells are particularly known for inhibiting CTL functions through the release of the anti-inflammatory IL-10, FGF-B and adenosine as well as the ‘consumption’ of IL-2 in the microenvironment, thereby reducing its availability for T cells. Combining cancer vaccines with molecules that can reduce the number of Treg cells, such as cyclophosphamide or low dose temozolomide (TMZ) thus constitutes another valid approach to improve the efficacy of anti-tumoral CTLs (126, 238, 239). Several studies indicate that 3–7 days after these chemotherapies may be the best timing to administer the cancer vaccine (84, 240, 241).

MDSCs represent another immunosuppressive leukocyte population frequently found in the tumor microenvironment which can limit the efficacy of anti-tumoral CTLs. Several myeloablative chemotherapeutics are known to decrease both peripheral and intra-tumor MDSCs, such as platinum salts, taxanes, gemcitabine, 5-Fluorouracil (242–244). The rationale of combining chemotherapy with therapeutic cancer vaccines to deplete MDSCs and boost vaccine-induced CTL responses has been reported in several studies (84, 238, 245). Specifically, in carboplatin-paclitaxel regimen the normalization of myeloid cells begins 2 weeks after the second cycle of chemotherapy and the administration of cancer vaccine at this point resulted in stronger vaccine-induced responses in preclinical and clinical studies (246–249).

Other studies reported improved anti-tumor responses also when chemotherapeutic agents were given simultaneously with the vaccination, as in the case of metronomic cyclophosphamide (170) or association of cyclophosphamide, paclitaxel and docetaxel (250). Given these results, the optimal schedule may be starting with chemotherapy cycles and following with concomitant chemotherapy and vaccination.

### Enhancing Cytotoxic T Lymphocyte Function by Combining Cancer Vaccines With Immune Checkpoint Inhibitors

Immune checkpoint inhibitors (ICIs), including antibodies against programmed cell death protein-1 (PD-1) or its ligand (PDL-1) and cytotoxic T-lymphocyte antigen-4 (CTLA-4) have proven to enhance anti-tumor immunity and efficacy in several cancers. However, a large subset of patients does not benefit from ICI therapy, with a reported objective response rate for anti-PD1 varying from almost absent (pancreatic cancer, glioma, microsatellite-stable colon adenocarcinoma) to 15–30% for most cancers, and 50–80% for few cancers including melanoma, Hodgkin lymphoma, squamous cell carcinoma and Merkel carcinoma (251). This low response rate observed in most cancers is likely related to a limited specific T cell response developed against cancer cells, especially for tumors with a low mutational burden. Therefore, combining a cancer vaccine, which can elicit specific T cell responses, and ICIs represents an attractive therapeutic option. Based on positive results in pre-clinical models (252–254), several clinical trials are now evaluating novel personalized vaccines against neoepitopes specific of each patient in combination with ICIs (NCT02950766, NCT02897765, NCT03289962) (**Figures 2B** and **3**). However, few studies address the point of the choice of a specific molecule. One preclinical study reported that anti-4-1BB antibody was superior to achieve anti-tumor efficacy in combination with peptide cancer vaccine compared with other immunomodulating antibodies (255). Also, the timing of combination therapy is rarely discussed in clinical trials, but some reports suggest that immune checkpoint inhibitors better synergize with the vaccine when administered at the time of the boost rather than at the prime (256).

### Combining Cancer Vaccine With Radiotherapy to Favor Antigen-Presentation by Cancer Cells

Numerous studies have shown the immunogenic properties of radiotherapy. Tumor irradiation can indeed induce immunogenic cell death (ICD) (257, 258) and thus lead to tumor regression even at distance sites, the so-called abscopal effect (259, 260). Additionally, radiotherapy has been reported to stimulate the expression of several molecules in cancer cells including MHC class I, death receptors, adhesion molecules, Fas, thus promoting CTL-mediated killing (261, 262). Therefore, combinatorial strategies of irradiation with the therapeutic cancer vaccine also constitute an attractive treatment option (263–265) (**Figures 2B** and **3**). However, radiotherapy is also responsible of reducing tumor infiltrating effector cells during the radiation regimen (266). Yet, the ultimate effect of radiotherapy synergizing with cancer vaccines is partly due to the vessel normalization that allows a better infiltration of T cells enhanced by the vaccine (267). Another preclinical study reported the best efficacy of vaccine when is administered 5 weeks after radiotherapy (268). In light of these findings, the combinatorial strategy of radiotherapy and cancer vaccine has more potential to succeed when radiotherapy is given first, followed by the vaccine (269).

## Understanding and Manipulating the Patients’ “Life-Style” to Increase Vaccine Potency

Although life expectancy has increased in Europe over the last 30 years, the so called “healthy life expectancy” has not, and many suffer from some form of chronic disease in the last 9–11 years of their life after the age of 65. In fact, 85% of deaths are caused by chronic diseases such as cancer, cardiovascular disease, chronic respiratory disease, diabetes, and mental illness, with 70 to 80% of healthcare costs being dedicated to the treatment and management of these conditions and diseases. Moreover, whereas cardiovascular diseases are the main cause of death after the age of 65, cancer remains the first and second cause of death before and after the age of 65 respectively. Risk factors known to be involved in chronic disease include repeat infections, obesity, diet, tobacco, radiation and environmental factors, all of which induce chronic disease through the induction of inflammation. Correctly regulated acute inflammation is the normal response to pathogens, irritants or damaged tissue, whereas chronic inflammation results from a failure to completely eliminate the pathogens, the inability to enzymatically remove the irritant, the body turning against self-proteins. However, chronic inflammation can also be the results of recurrent acute inflammation. In recent years, the importance of the microbiota has been revealed, including alterations during chronic inflammation. Furthermore, more recent work has highlighted how a disturbed microbiota cannot only play a part in exacerbating inflammation but can drive the process. For example, in immunotherapy against cancer, studying a patient’s intestinal microbiota composition can be used to stratify patients into “responders” *versus* “non-responder” according to their intestinal microbiota composition (270, 271). Indeed, in the study from V. Gopalakrishnan et al., 2018 (271), patients with metastatic melanoma who responded to anti-PD1 treatment, with longer progression-free survival, were found to have a higher diversity of bacteria as well as a significantly higher number of the *Ruminococcaceae* family in their fecal microbiota. Interestingly, the prevalence of this family of bacteria increases during alcohol abstinence and inversely correlates with intestinal permeability (272). This species has also been shown to have *in vitro* and *in vivo* anti-inflammatory properties (273). Importantly, similar studies in mice have demonstrated that response to treatment could be transferred from responders to non-responders *via* fecal transplantation into tumor-bearing germ-free mice (271). However, other studies have shown the importance of other bacteria such as *Bifidobacterium longum*, *Collinsella aerofaciens*, and *Enterococcus faecium*, and *Akkermansia muciniphila* the latter being systematically found in higher number in patients with advanced melanoma who respond to anti-PD-L1 treatment (274), whereas patients with advanced urothelial carcinoma, non-small cell lung cancer, and renal cell carcinoma who received antibiotics before, during and after treatment all experienced reduced progression-free survival and lower overall survival rates, thereby demonstrating the importance of not disturbing the microbiota (275).

Most cancer vaccines, including cancer vaccines, will require some form of adjuvant to either induce/boost a response, increase the speed of the response, allow for a more reduce dose to be used and/or reduce the number of immunizations. In view of the importance/influence the microbiota on a person’s overall wellbeing and the immune system in particular, it is of prime importance to understand ways to improve this biodiversity, as well as to increase the number of “beneficial” bacteria present in the patient’s intestinal microbiota before, during and after vaccination. Increasing the diversity of bacteria within the intestinal flora has been shown to improve metabolic and immunological functions (276). No clear data is available about cancer vaccine, but the efficacy of vaccination against several pathogen has been clearly correlated with microbiota. Microbiota can indeed act as a natural vaccine adjuvant and specifically as ligand for different TLRs. Flagellin (TLR5 ligand) from microbiota seems to play a crucial role since levels of TLR5 have been correlated to the magnitude of humoral response (277). Recently, microbiota has also been reported to enhance anti-tumor response when used as a real cancer vaccine adjuvant in a murine model [EGFR vIII-expressing Listeria vaccine, (278)].

In light of these results, the use of probiotics, or novel genetically modified bacteria, may improve the efficacy of cancer vaccine. In addition, the microbiota is sensitive and will respond to physiological changes taking place in the host due to internal and external factors such as lifestyle, exercise, diet and the physiology of the host and this, in turn, will influence the well-being of the host. Exercise has already been shown to have a role in reducing the risk of cancer, and to be associated with a lower incidence of cancer and a lower risk of recurrence (279, 280). These effects and associations have been linked with the ability of exercise to influence immune cells such as NK, T cells, B cells and DCs, all of which have been found at a higher density within the tumors of animals who had been allowed to freely use an exercise wheel (281). Out of all these cells, NK cells (which express the highest number of β-adrenergic receptors) were the most sensitive to exercise, in that they were recruited within minutes after the start of exercise (282). These effects were shown to be driven, at least in part, by exercise-induced increases in catecholamine production (282). Moreover, the relationships between the hypothalamic-pituitary-adrenal (HPA) axis, the autonomic nervous system and the immune system and its effect on the microbiota have previously been neglected and certainly never been taken into account prior to, or during cancer vaccine treatment. Yet, this Gut-Brain axis is bi-directional whereby gut-microflora and brain communicate and are influenced by each other’s signals *via* neural, endocrine, humoral and immune links. Therefore, as highlighted above, activities such as exercise which increases the level of neurotransmitters such as catecholamines, and the consumption of certain food such dietary fibers which will increase the production of short chain fatty acids such as butyrate, generated by anaerobic bacteria during fermentation which in turn will influence the production of neuropeptide such as NPY, will have a significant impact on the activation or suppression of certain immune cells. Butyrate itself is a histone deacetylase inhibitor that has been shown to suppress tumor growth (283–285). NPY receptors are widely expressed on immune cells, especially Y1R, which exists on almost every type of immune cells, and have an important yet diverse role on the immune system, having both negative as well as activator functions (286) [For a full review on the immunomodulatory activity of NPY please read Chen et al. (287)]. Targeting selectively certain neuropeptide receptors will therefore open more drug development to improve vaccine potency as well as offer novel vaccine deliver system.

NPY levels often increase during stress responses, and NPY receptors are shown to be overexpressed by many well-innervated cancers such as prostate cancer, the trans-differentiation of which into aggressive neuroendocrine prostate cancer (NEPC) after a long period of androgen-deprivation-therapy (ADT) treatments often leads to metastasis progression and incurable disease. NEPC expresses high levels of β2-adrenergic receptors (ADRs) which can be activated by adrenergic signals triggered by depression or chronic stress, which is prevalent in men with prostate cancer. Improving the efficacy of immunotherapies will therefore require approaches to attenuate the immunosuppressive nature of the tumor microenvironment (TME), increase the biodiversity and the number of “good” bacteria as well take into account the impact of depression and chronic stress.

Although the precise mechanisms underlying such intricate connections are only now starting to be elucidated there is absolutely no doubt that they will need to be carefully assessed if one wants to achieve optimum vaccine efficacy. However, most of the scientific vaccine community is now focused on the microbiota, forgetting the rest of the axis.

Therefore, future successful cancer therapy as well as vaccination strategies may be those that approach the therapy using both an effective vaccine but also include therapeutic strategies that influence the life-style impacting the immune system.

## Conclusions

Therapeutic vaccines represent an attractive strategy to stimulate the immune system against cancer in combination with standard therapies. However, multiple cancer vaccines have not yet achieved significant clinical efficacy. Their limited efficacy is certainly in part related to the poor immunogenicity of the vaccine itself in many cases, but also to the difficulty of inducing an effective immune response in the compromised immune system of cancer patients. Indeed, cancer cells successfully grow by establishing an immunosuppressive tumor microenvironment to protect themselves from host’s immune attack. To add to this the median age of cancer patients is 66 years old and the immune system is known to become less efficient and more dysregulated as people age. However, while on the one hand the immune system declines over time, a phenomenon known as immunosenescence, aging is also known to be associated with a low, but persistent level of inflammation, inflamm-aging, which also leads to dysregulation of innate and adaptive immune cells. Therefore, the choice of adjuvants in vaccine formulations needs careful optimization for each vaccine as well as for each patient if the maximum immune response and clinical efficacy in such a compromised condition is to be achieved. Globally, given the crucial role of CD8+ T cells in tumor control, adjuvants capable of eliciting cellular response, rather than humoral, are certainly preferable. Indeed, levels of CD8+ T cells induced after cancer vaccine have been correlated with tumor regression in both murine and clinical studies (256, 288). More specifically, promising adjuvants are those that proved to favor dendritic cells maturation (the principal APC in tumor context) and cross-presentation. Among the formers, STINGs and TLR agonists (especially CpG, albeit more in mice than in human and poly I:C) demonstrated the most encouraging results. The induction of DC maturation is in fact a crucial point in vaccine strategy to avoid self-antigens tolerance. In addition, given the defects on DCs described in elderly individuals, TLR ligands likely represent the most promising immunomostimulatory adjuvant candidates for cancer vaccines in these patients. Beside the maturation of DCs, the ideal vaccine formulation should also favor the cross-presentation of antigens to CD8+ T cells by DCs. In that respect, several vectors are under development. Although live vectors from virus or bacteria can efficiently induce cross-presentation of antigens, the vector itself being immunogenic, elicits an immune response. Therefore, after the first dose of vaccine, the subsequent boost doses need to use different vectors in order to overcome the neutralization of vectorized vaccine by host immunity. Consequently, this approach has limited prospects in clinical practice. Other not live vectors showed interesting results in mice, such as liposomes, virosomes or nanoparticles. They have the advantage of being able to deliver different source of antigens (RNA, DNA, proteins, peptides, …) and adjuvants and also be immunostimulatory by assembling both molecules in a package and carry it to secondary lymphoid organs. Compared to classic depot adjuvants such as IFA or MF59, these vectors, allow antigens to reach directly the lymph nodes in order to induce a more efficient cross-presentation between DCs and T cells. The use of these adjuvants may thus overcome the detrimental effect that some study reported for W/O emulsions, related to the persistent release of antigen and the inflammation in the injection site. In fact, if in one hand the slow release of antigen may promote a stronger immune response, on the other hand it can lead to T cell anergy if DCs are immatures or T cell sequestration at the injection site has occurred. However, although very promising, vector adjuvants have not yet demonstrated convincing efficacy in humans.

In the light of these results, not-depot adjuvants are thus preferable, but the schedule of this particular type of vaccination is still a crucial point, and unfortunately not directly addressed in clinical trials and only rarely in pre-clinal studies. A too short period between boost and priming vaccinations might indeed lead to immunological tolerance against the antigens. In a mouse model, Wick *et al*. reported a decline of response from 30% to 15% (circulating specific T cells) by day 10 of daily vaccinations with a formulation using poly I:C and OVA protein (289). Another study by Stark *et al*. showed similar results in a B16-OVA melanoma model using a vaccine formulation with archae liposoms (archaeosomes) (290). In this study, the authors vaccinated mice with a regular interval of 0, 21, 42, 72 and 110 days and a decline of response was seen after the third dose. However, beside the potency of immunological response (amount of specific T cells) induced at the time of vaccination, it is also important to achieve a prolonged tumor protection. This latter has been particularly correlated with a central memory phenotype (CD62L^high^) of vaccine-induced T cells, rather than effector memory (CD62L^low^). In that respect, interestingly, in the prophylactic model of Stark *et al*. even if a single dose of vaccine triggered a lower frequency of antigen-specific CD8 T cells than multiple doses, the late tumor protection was similar (tumor challenge on day 323). These results highlight the importance of the quality of the immunological response besides the quantity.

Lastly, different combinatorial approaches are being explored trying to enhance the efficacy of the vaccine. Despite the numerous encouraging results in pre-clinical studies, clinical responses to cancer vaccine as monotherapy have been rather anecdotal so far and they are mostly reported in case of pre-neoplastic lesions or low tumor burden. However, the goal of a therapeutic cancer vaccine should be to improve survival in patients with advanced cancers. In this common situation, the immune response elicited by the vaccine needs to be particularly strong to face the suppressive nature of the tumor microenvironment and more generally the immunocompromised system of the patients in this context, associating different adjuvants, especially an immunostimulatory molecule with a vector adjuvant, may certainly improve the efficacy of the vaccine. In addition, combining cancer vaccine with other treatments is more likely to succeed, but early intervention may also be of value. Several combinatorial strategies are being explored, such as with anti-angiogenic treatments to promote T cell homing to tumors, with immune checkpoint inhibitors to enhance CTL function, and also with standard anti-cancer therapies such as chemotherapy to deplete immunosuppressive leucocyte populations, and radiotherapy to favor antigen-presentation by cancer cells. As mentioned, a crucial point, rarely addressed in clinical trials, is the optimal timing of such therapies. In the light of the synergic mechanism specifically involved, therapies acting through the clearance of immunosuppressive cells such as radiotherapy and chemotherapy should be given prior to vaccination, whereas immunostimulatory agents enhancing the anti-tumor response of the vaccine should be administered concomitantly with the vaccine (preferentially at the boost dose) as in the case of immune checkpoint inhibitors. It may also be favorable to use vaccines in combination with early surgical intervention as the size of the lesion may hamper effective infiltration into the tumor. If the early intervention is used, then therapy effects could be achieved without a risk for immune suppression. Also, the host is less likely to have been negatively impacted by the tumor, immobilization and/or toxic drugs, making the patient more likely to still have a healthy and functional immune system.

In addition to these more traditional approaches, more clinical trials should consider implementing changes in the diet/exercise/stress level of the patients, while the patients are recovering from other more aggressive form of treatments (chemotherapy, radiotherapy) in order to re-establish a functional more effective immune system prior to the administration of a vaccine and carefully monitor the effect these will have on the diversity/quantity of their microbiome and their immune status, before and after vaccination in order to assess their impact and overall benefit for the patients.

## Author Contributions

SC, KH, and SMc wrote the initial draft, with DN, SMa, and AP making significant contribution. All authors discussed and reviewed the content.

## Conflict of Interest

SMa is the Chief Development Officer at Ultimovacs AB, a company that develops cancer vaccines and holds patent applications within the field of cancer vaccines. SMa is also the founder of Immuneed AB and Vivologica AB. AP is the Chief Executive Officer of multimmune GmbH, a company that develops cancer “theranostics” based on tumor expression of membrane Hsp70.

The remaining authors declare that the research was conducted in the absence of any commercial or financial relationships that could be construed as a potential conflict of interest.
